# Association and interaction of *APOA5*, *BUD13*, *CETP*, *LIPA* and health-related behavior with metabolic syndrome in a Taiwanese population

**DOI:** 10.1038/srep36830

**Published:** 2016-11-09

**Authors:** Eugene Lin, Po-Hsiu Kuo, Yu-Li Liu, Albert C. Yang, Chung-Feng Kao, Shih-Jen Tsai

**Affiliations:** 1 Graduate Institute of Biomedical Sciences, China Medical University, Taichung, Taiwan; 2 Vita Genomics, Inc., Taipei, Taiwan; 3 TickleFish Systems Corporation, Seattle, WA, USA; 4 Department of Public Health, Institute of Epidemiology and Preventive Medicine, National Taiwan University, Taipei, Taiwan; 5 Center for Neuropsychiatric Research, National Health Research Institutes, Miaoli County, Taiwan; 6 Department of Psychiatry, Taipei Veterans General Hospital, Taipei, Taiwan; 7 Division of Psychiatry, National Yang-Ming University, Taipei, Taiwan; 8 Department of Agronomy, College of Agriculture & Natural Resources, National Chung Hsing University, Taichung, Taiwan

## Abstract

Increased risk of developing metabolic syndrome (MetS) has been associated with the *APOA5*, *APOC1*, *BRAP*, *BUD13*, *CETP*, *LIPA*, *LPL*, *PLCG1*, and *ZPR1* genes. In this replication study, we reassessed whether these genes are associated with MetS and its individual components independently and/or through complex interactions in a Taiwanese population. We also analyzed the interactions between environmental factors and these genes in influencing MetS and its individual components. A total of 3,000 Taiwanese subjects were assessed in this study. Metabolic traits such as waist circumference, triglyceride, high-density lipoprotein (HDL) cholesterol, systolic and diastolic blood pressure, and fasting glucose were measured. Our data showed a nominal association of MetS with the *APOA5* rs662799, *BUD13* rs11216129, *BUD13* rs623908, *CETP* rs820299, and *LIPA* rs1412444 single nucleotide polymorphisms (SNPs). Moreover, *APOA5* rs662799, *BUD13* rs11216129, and *BUD13* rs623908 were significantly associated with high triglyceride, low HDL, triglyceride, and HDL levels. Additionally, we found the interactions of *APOA5* rs662799, *BUD13* rs11216129, *BUD13* rs623908, *CETP* rs820299, *LIPA* rs1412444, alcohol consumption, smoking status, or physical activity on MetS and its individual components. Our study indicates that the *APOA5*, *BUD13*, *CETP*, and *LIPA* genes may contribute to the risk of MetS independently as well as through gene-gene and gene-environment interactions.

The metabolic syndrome (MetS), a chronic and complex disease, is characterized by having large waist circumference plus two or more of the following factors: raised triglyceride levels, low high-density lipoprotein (HDL) cholesterol levels, raised blood pressure, and raised glucose levels^1^. Due to escalating prevalence rates and its risk for the development of several chronic complications such as cardiovascular diseases, MetS has become a major public health challenge in Taiwan and at the global scale^2^. MetS is primarily caused by a combination of genetics and environmental factors such as health-related behaviors^2^,^3^. While more and more MetS risk loci have been identified, it has long been noted that genetic variants conferring susceptibility may vary across ethnicities^4^. Among the genes involved in the development of MetS and/or cardiovascular diseases are the apolipoprotein A5 (*APOA5*), apolipoprotein C1 (*APOC1*), BRCA1 associated protein (*BRAP*), BUD13 homolog (*BUD13*), cholesteryl ester transfer protein (*CETP*), lipase A lysosomal acid type (*LIPA*), lipoprotein lipase (*LPL*), phospholipase C gamma 1 (*PLCG1*), and ZPR1 zinc finger (*ZPR1*) gene.

The *APOA5* gene is located on chromosome 11q23 and encodes an apolipoprotein protein that has been implicated in regulating the plasma triglyceride levels, a major risk factor for coronary artery disease (CAD). A common single nucleotide polymorphism (SNP), rs662799 (−1131T > C), located in the promoter region of the *APOA5* gene is one of the most extensively studied variants. The relationship between the MetS and the *APOA5* rs662799 SNP has been ambiguous. The *APOA5* rs662799 SNP has been reported to increase the risk of acquiring MetS in Caucasians^5^ and in Asians residing in Japan^6^, Taiwan^7^, Hong Kong^8^, China^9^, and Korea^10^. In contrast, this association has not been replicated in Caucasian^11^,^12^,^13^, Arabic^14^, and Hispanic^15^ populations. Several meta-analysis studies have also suggested that the *APOA5* rs662799 SNP is associated with an increased risk of developing MetS in Asians, but not in European populations^9^,^16^.

Furthermore, the *LIPA* gene is located on chromosome 10q23 and encodes the lysosomal acid lipase enzyme, which functions in the lysosome of cells to hydrolyze cholesteryl esters and triglycerides and then to generate free cholesterol and free fatty acids. Several genome-wide association studies (GWAS) indicated that the rs1412444 SNP in the intron region of the *LIPA* gene was associated with CAD in Caucasian and Asian populations^17^,^18^. Evidence has also been reported for an association of *LIPA* rs1412444 with MetS^19^, with premature CAD^19^, and with myocardial infarction^20^ in independent replication studies. In addition, Kraja *et al.* performed a GWAS study on data from 7 cohorts in Caucasian populations and detected a significant association of MetS with the *APOA5*, *BUD13*, *CETP*, *LPL*, and *ZPR1* genes^21^. The following GWAS study by Avery *et al.* indicated that the *APOC1*, *BRAP*, and *PLCG1* genes may contribute to the susceptibility for MetS in European Americans and African Americans^22^. Moreover, another GWAS study by Kristiansson *et al.* implicated that the *ZPR1* gene may be involved with MetS susceptibility in Finnish cohorts^23^.

Given that gene-gene interactions may play a key role in the development of MetS, we hypothesized that SNPs within the aforementioned genes including *APOA5*, *APOC1*, *BRAP*, *BUD13*, *CETP*, *LIPA*, *LPL*, *PLCG1*, and *ZPR1* may contribute to the etiology of MetS and its individual components independently and/or through complex interactions. Furthermore, the interplay between SNPs within these genes and health-related behaviors, such as alcohol consumption, smoking status, and physical activity, has not been fully evaluated in previous association studies. In light of the aforementioned considerations, we thus assessed both the primary effects of single loci and multilocus interactions for an association of SNPs within these genes with the prevalence of MetS and its individual components in Taiwanese individuals. We also determined whether significant gene-environment interactions exist between SNPs within these genes and health-related behaviors.

## Results

Table 1 describes the demographic and clinical characteristics of the study population, including 533 MetS subjects and 2,467 non-MetS subjects. The MetS prevalence in our cohort was 17.8%. As shown in Table 1, the distribution of gender was well matched, and the distribution of age was not matched. Moreover, there was a significant difference in waist circumference, triglyceride, HDL, blood pressure, and fasting glucose between the MetS and non-MetS subjects (Table 1; all P < 0.0001, respectively). Furthermore, there was a significant difference in current alcohol consumption (P = 0.029) and smoking status (P = 0.001) between the MetS and non-MetS subjects. However, there were no significant differences found between participants with and without the MetS in level of physical activity.

Among the 82 SNPs investigated in this study (Supplementary Table S1), there were 19 SNPs showing an evidence of association (P < 0.05) with MetS. However, none of the SNPs were significantly associated with MetS after Bonferroni correction (P < 0.05/82 = 0.0006). We also calculated pairwise linkage disequilibrium (LD) between 82 SNPs, and Supplementary Table S2 shows a list of SNP pairs with strong LD (r^2^ > 0.8). As shown in Table 2, we then selected the five key SNPs (including *APOA5* rs662799, *BUD13* rs11216129, *BUD13* rs623908, *CETP* rs820299, and *LIPA* rs1412444) with nominal evidence of association (P < 0.01), which were further examined in the subsequent analyses. In addition, the genotype frequency distributions for the *APOA5* rs662799, *BUD13* rs11216129, *BUD13* rs623908, *CETP* rs820299, and *LIPA* rs1412444 SNPs were in accordance with the Hardy–Weinberg equilibrium among the subjects (P = 0.595, 0.762, 0.692, 0.278, and 0.245, respectively).

Moreover, the OR analysis showed risk genotypes of variants of *APOA5* rs662799, *BUD13* rs11216129, *BUD13* rs623908, *CETP* rs820299, and *LIPA* rs1412444 after adjusting for covariates, indicating an increased MetS risk among the subjects (Table 2). As demonstrated in Table 2 for the *CETP* rs820299 SNP, there was an indication of an increased MetS risk among the MetS and non-MetS subjects after adjustment of covariates such as age, gender, smoking, alcohol consumption, and physical activity for genetic models, including the recessive model (OR = 1.47; 95% CI = 1.16–1.86; P = 0.0015) and additive model (OR = 1.17; 95% CI = 1.02–1.34; P = 0.0211). Similarly, there was an indication of an increased risk of MetS among the subjects after adjustment of covariates for genetic models in the *APOA5* rs662799 (P [additive model] = 0.0086; P [recessive model] = 0.0229; P [dominant model] = 0.0218), *BUD13* rs11216129 (P [dominant model] = 0.0027), *BUD13* rs623908 (P [dominant model] = 0.0027), and *LIPA* rs1412444 (P [additive model] = 0.0097; P [recessive model] = 0.0171) SNPs (Table 2). Additionally, there were still residual associations between MetS and *APOA5* rs662799 (P = 0.0114) as well as between MetS and *CETP* rs820299 (P = 0.0399) after further accounting for triglyceride and HDL, suggesting an independent association of MetS with *APOA5* rs662799 and *CETP* rs820299.

Next, Table 3 shows the analysis of the *APOA5* rs662799, *BUD13* rs11216129, *BUD13* rs623908, *CETP* rs820299, and *LIPA* rs1412444 SNPs with the individual components of MetS (as quantitative measures) including waist circumference, triglyceride, HDL, systolic blood pressure, diastolic blood pressure, and fasting glucose. When we treated the phenotypes as quantitative measures rather than dichotomous ones, there was evidence of an association between these five SNPs and quantitative traits such as triglyceride, HDL, or fasting glucose (Table 3). As shown in Table 3 for the *APOA5* rs662799, *BUD13* rs11216129, and *BUD13* rs623908 SNPs, there was a significant difference in triglyceride or HDL (after Bonferroni correction; P < 0.0006) among the subjects after adjustment of covariates for genetic models.

In addition, the GMDR analysis was used to assess the impacts of combinations between the five SNPs in MetS and its individual components including age, gender, smoking, alcohol consumption, and physical activity as covariates. Table 4 summarizes the results obtained from GMDR analysis for two-way up to five-way models with covariate adjustment. As shown in Table 4 for MetS, there was a significant two-way model involving *CETP* rs820299 and *LIPA* rs1412444 (P = 0.005), indicating a potential gene-gene interaction between *CETP* and *LIPA* in influencing MetS. The effect of *CETP* rs820299 and *LIPA* rs1412444 interaction remained significant after Bonferroni correction (P < 0.05/5 = 0.01). The *CETP* rs820299 and *LIPA* rs1412444 interaction was shown to be statistically significant (OR = 1.26; 95% CI = 1.02–1.54; P = 0.0282) in the subsequent logistic regression analysis, adjusted to age, gender, smoking, alcohol consumption, and physical activity. Further, our analysis suggested that the individuals carrying the risk allele for *CETP* rs820299 were more likely to also carry the risk alleles for *LIPA* rs1412444 (P = 0.05). Additionally, there were a three-way model involving *BUD13* rs623908, *CETP* rs820299, and *LIPA* rs1412444 (P = 0.001) as well as a four-way model involving *APOA5* rs662799, *BUD13* rs623908, *CETP* rs820299, and *LIPA* rs1412444 (P = 0.012), indicating a potential gene-gene interaction among *APOA5*, *BUD13*, *CETP*, and *LIPA* in influencing MetS. The effect of the three-way model remained significant after Bonferroni correction (P < 0.01); however, the effect of the four-way model did not. Similarly, there were significant two-way up to four-way gene-gene interaction models (P < 0.001) in influencing individual components such as high triglyceride or low HDL, and the effect remained significant after Bonferroni correction (P < 0.01).

Moreover, Table 5 shows the GMDR analysis of gene-environment interaction models in MetS and its individual components using age and gender as covariates. As shown in Table 5 for MetS, there were a significant two-way model involving *BUD13* rs623908 and smoking (P < 0.001), a three-way model involving *BUD13* rs623908, *CETP* rs820299, and smoking (P < 0.001), a four-way model involving *BUD13* rs623908, *CETP* rs820299, *LIPA* rs1412444, and smoking (P < 0.001), as well as a five-way model involving *BUD13* rs623908, *CETP* rs820299, *LIPA* rs1412444, smoking, and physical activity (P < 0.001), indicating a potential gene-environment interaction among *BUD13*, *CETP*, *LIPA*, smoking, and physical activity in influencing MetS. The effect of these models remained significant after Bonferroni correction (P < 0.05/8 = 0.006). Similarly, there were significant two-way up to five-way gene-environment interaction models in influencing individual components such as high triglyceride (P < 0.001) or low HDL (P < 0.001), and the effect remained significant after Bonferroni correction (P < 0.006).

Furthermore, we utilized multivariable logistic regression analysis with adjustment for age and gender to assess the two-way gene-environment interaction models selected by the GMDR method (Supplementary Table S3). Our analysis revealed that smokers with the G allele of *BUD13* rs623908 had a 1.61-fold increased risk for MetS, compared to non-smokers with the AA genotype of *BUD13* rs623908 (Supplementary Table S3). Similarly, smokers with the C allele of *APOA5* rs662799 had a 3.42-fold increased risk for high triglyceride, compared to non-smokers with the TT genotype of *APOA5* rs662799 (Supplementary Table S3). Additionally, smokers with the C allele of *APOA5* rs662799 had a 2.62-fold increased risk for low HDL, compared to non-smokers with the TT genotype of *APOA5* rs662799 (Supplementary Table S3). Moreover, individuals with the G allele of *CETP* rs820299 and low levels of physical activity had a 1.44-fold increased risk for high waist circumference, compared to those with the A allele of *CETP* rs820299 and high levels of physical activity (Supplementary Table S3).

Finally, statistical power analysis revealed that the present study had a 99.9% power to detect associations of *APOA5* rs662799 (effect size = 1.26; minor allele frequency (MAF) = 27.2%), *BUD13* rs11216129 (effect size = 0.74; MAF = 26.8%), *BUD13* rs623908 (effect size = 0.75; MAF = 30.8%), *CETP* rs820299 (effect size = 1.47; MAF = 41.7%), or *LIPA* rs1412444 (effect size = 1.22; MAF = 32.7%) with MetS among the MetS and non-MetS subjects after applying Bonferroni correction (P < 0.0006).

## Discussion

Our replication study is the first study to date to examine whether the main effects of the *APOA5*, *APOC1*, *BRAP*, *BUD13*, *CETP*, *LIPA*, *LPL*, *PLCG1*, and *ZPR1* genes are significantly associated with the risk of MetS and its individual components independently and/or through gene-gene interactions among Taiwanese individuals. We also investigated the relationship between these genes and health-related behaviors to examine whether these genes confer a risk of MetS according to its effect on gene-environment interactions. In this study, we found that *APOA5* rs662799, *BUD13* rs11216129, *BUD13* rs623908, *CETP* rs820299, and *LIPA* rs1412444 were linked with MetS. Additionally, our data revealed that *APOA5* rs662799, *BUD13* rs11216129, and *BUD13* rs623908 were significantly associated with the individual components of MetS such as high triglyceride and low HDL (as well as with triglyceride and HDL levels). Our data also indicated that gene-gene interactions of *APOA5*, *BUD13*, *CETP*, and *LIPA* may contribute to the etiology of MetS. Finally, there was a significant gene-environment interaction between these four genes and health-related behaviors, such as alcohol consumption, smoking status, and physical activity.

Here, we report for the first time that the *BUD13* rs11216129, *BUD13* rs623908, and *CETP* rs820299 SNPs may play an important role in the modulation of MetS in a Taiwanese population. In addition, we observed that there were a significant association of *BUD13* rs11216129 and *BUD13* rs623908 with high triglyceride and low HDL as well as a significant association of both SNPs with triglyceride and HDL levels. Our data also suggested that *CETP* rs820299 was involved in high waist circumference, high triglyceride, and HDL levels. Similarly, previous studies reported that *BUD13* rs10790162 21, *CETP* rs173539 21, and *CETP* rs708272 24 may contribute to the susceptibility for MetS in European subjects^21^ and Mexican women^24^. However, we did not detect an association between *BUD13* rs10790162 and MetS in the present study. Further, we did not test *CETP* rs173539 and *CETP* rs708272 due to lack of these two SNPs in the custom chip. Previously, the *CETP* gene has been reported in association with HDL levels in Caucasian^21^,^25^,^26^ and Asian Indian^25^ subjects as well as with higher triglyceride levels in Caucasian subjects^21^. Additionally, *BUD13* variants have been associated with triglyceride levels^27^,^28^, total cholesterol levels^27^, and hypercholesterolaemia^29^ in Chinese subjects.

Moreover, another intriguing finding was a positive association of *LIPA* rs1412444 with MetS, low HDL, high fasting glucose, HDL levels, and fasting glucose levels in a Taiwanese population. In line with our results, a previous study by Vargas-Alarcón *et al.* demonstrated that the *LIPA* rs1412444 polymorphism was likely to influence MetS and hypertriglyceridemia in a Mexican population^19^. It has also been suggested that the *LIPA* rs1412444 polymorphism was involved in CAD^17^,^18^, premature CAD^19^, and myocardial infarction^20^. Furthermore, Wild *et al.* reported a strong association of the CAD risk allele (T) of *LIPA* rs1412444 with higher *LIPA* expression as well as an association of elevated *LIPA* expression with lower HDL levels and subclinical atherosclerotic disease^30^. Additionally, mutations in the *LIPA* gene are the cause of Wolman’s Disease, Cholesteryl ester storage disease, hyperlipidemia, premature cardiovascular disease, and increased risk for atherosclerosis^31^. Finally, it should be noted that the T allele frequency of *LIPA* rs1412444 varies considerably between different ethnic populations, ranging from 34% in European subjects^17^, 51% in South Asian subjects^17^, 49.1% in Mexican subjects^19^, 32% in Chinese subjects^20^, 32.5% in German subjects^30^, and 32.7% in the present Taiwanese population assessed in our study.

The *APOA5* rs662799 polymorphism has been widely implicated to affect the MetS risk^9^, although genetic evidence of its effect on MetS has been inconsistent. In this study, we observed that there was an association of *APOA5* rs662799 with MetS after covariate adjustment in OR analysis. Our results are in agreement with those of several other studies^5^,^6^,^7^,^8^,^9^,^10^. We also observed that there was a significant association of *APOA5* rs662799 with high triglyceride and low HDL as well as with triglyceride and HDL levels. Xu *et al.* performed a meta-analysis on data from 91 studies including 51,868 subjects in Asian, European, and other ethnic populations and detected a significant association of the C allele of *APOA5* rs662799 with elevated triglyceride levels and decreased HDL levels^9^. In the subgroup analysis stratified by the ethnicity, this association was also detected in both Asian and European populations^9^. It is worth mentioning that the C allele frequency of *APOA5* rs662799 varies considerably between different ethnic populations, ranging from 8.5% in Hungarian subjects^5^, 35.3% in Japanese subjects^6^, 28.6% in Hong Kong subjects^8^, 21.6% in Chinese subjects^9^, 7% in Germany subjects^11^, and 27.2% in the present Taiwanese population assessed in our study.

By using the GMDR approach, we further inferred the epistatic effects between *APOA5*, *BUD13*, *CETP*, and *LIPA* in influencing MetS and its individual components. To our knowledge, no other study has been conducted to evaluate gene-gene interactions between these genes. Although *ZPR1* was not a key gene in the present study (that is, no association with MetS), Aung *et al.* identified a potential gene-gene interaction between the *BUD13* and *ZPR1* genes on the risk of hypercholesterolaemia and hypertriglyceridaemia in Chinese subjects using GMDR analyses^29^. Another promising finding in the present study was an interaction between these genes and environmental factors in MetS and its individual components. In accordance with our analysis, Wu *et al.* reported that *APOA5* rs662799 had a positive interaction with environmental factors, such as tobacco use and alcohol consumption, on MetS with participations in China^32^. Likewise, a previous study by Hiramatsu *et al.* found the synergistic effects of *APOA5* rs662799 and the rs6929846 SNP of the butyrophilin subfamily 2 member A1 (*BTN2A1*) gene on the development of MetS in Japanese individuals^33^. Son *et al.* also suggested an interaction between *APOA5* rs662799 and alcohol drinking as well as an interaction between *APOA5* rs662799 and physical activity in affecting triglyceride levels in Korean men, but no interaction between *APOA5* rs662799 and smoking status^34^.

While our results showed that the individuals carrying the G allele of *BUD13* rs623908 had a protective effect (OR = 0.75) for MetS (as compared to those carrying the AA genotype of *BUD13* rs623908), the interaction effect between *BUD13* rs623908 and smoking on MetS yielded an OR value of 1.61 when we compared smokers carrying the G allele of *BUD13* rs623908 with non-smokers carrying the AA genotype of *BUD13* rs623908. Our analysis also implicated the interaction effect between *APOA5* rs662799 and smoking on high triglyceride (OR = 3.42) or low HDL (OR = 2.62) as well as the interaction effect between *CETP* rs820299 and physical activity on high waist circumference (OR = 1.44). According to our and previous results^32^, smoking seems to cause increased health risks, especially for the individuals with the CT and CC genotypes of *APOA5* rs662799.

Besides the statistical significance, the potential biological mechanism under the interaction models was our concern. The functional relevance of the interactive effects of *APOA5*, *BUD13*, *CETP*, and *LIPA* on MetS remains to be elucidated. If there is a deficiency of lysosomal acid lipase encoded by the *LIPA* gene, lipids such as triglycerides and cholesteryl esters accumulate in the cell, resulting in pre-mature atherosclerosis^35^. It has also been suggested that *LIPA* rs1412444 is associated with increased *LIPA* expression, which is expected to enhance intracellular release of fatty acids and cholesterol via the lysosomal route^30^,^35^. Furthermore, the risk allele of *LIPA* rs1412444 may increase the generation of free cholesterol in the arterial intima and, likely as a consequence, may promote an inflammatory process and atherosclerotic plaque formation^30^. Likewise, it is speculated that *APOA5* rs662799 may be involved in the regulation of gene transcription due to its location in the promoter region and thereby considerably impact serum apolipoprotein A5 levels^6^. Additionally, an animal study showed that overexpression of human *APOA5* in mice is correlated with decreased plasma triglyceride levels^36^. Moreover, *APOA5*, *BUD13*, and *CETP* are known to play a key role in lipid metabolism^21^. Some speculate that the association of the *BUD13* gene with serum lipid levels may be relevant to the nearby *APOA5* gene because *BUD13* is located in the downstream of *APOA5*^29^. Finally, *CETP* contributes to lower HDL since it enables the transfer of cholesteryl esters in HDL toward triglyceride-rich lipoproteins^21^.

This study has both strengths and limitations. The main weakness of this study is that our observations require much further validation to test whether the findings are replicated in various ethnic groups^37^,^38^. Second, to our knowledge, there are no viable molecular biological models that support the gene-gene and gene-environment interactions found in this study. In future work, prospective clinical trials with other ethnic populations are necessary to facilitate a thorough evaluation of the association and interactions of the investigated SNPs with MetS and its individual components^39^,^40^. On the other hand, an important strength of our study was the use of health-related behavior data, which provided a unique opportunity to examine the interactions between the investigated polymorphisms and health-related behaviors.

In conclusion, we carried out an extensive analysis of the association as well as gene-gene and gene-environment interactions of the *APOA5*, *BUD13*, *CETP*, and *LIPA* genes with MetS and its individual components in Taiwanese subjects. Our findings demonstrate that the *APOA5*, *BUD13*, *CETP*, and *LIPA* genes may affect the prevalence of MetS independently and/or through complex gene-gene and gene-environment interactions. Furthermore, the *APOA5* and *BUD13* genes are a determinant of MetS component factors, such as high triglyceride and low HDL. Independent replication studies with larger sample sizes will likely provide further insights into the role of the *APOA5*, *BUD13*, *CETP*, and *LIPA* genes found in this study.

## Materials and Methods

### Study population

This study incorporated subjects from the Taiwan Biobank^41^. The study cohort consisted of 3,000 participants. Recruitment and sample collection procedures were approved by the Internal Review Board of the Taiwan Biobank before conducting the study. Each subject signed the approved informed consent form. All experiments were performed in accordance with relevant guidelines and regulations.

Current alcohol drinker was defined as currently drinking 150 ml of alcohol per week for more than six months. Current smoker was defined as currently smoking for more than six months. Physical activity was defined by the amount of excise activity for more than three times and more than 30 minutes each time in each week.

### Metabolic Syndrome

The MetS was diagnosed using the International Diabetes Federation (IDF) definition, which requires that the participant represented by central obesity (defined as waist circumference ≥90 cm in male subjects and ≥80 cm in female subjects) plus the presence of two or more of the following four components: (1) triglycerides ≥150 mg/dl; (2) HDL cholesterol <40 mg/dl in male subjects and <50 mg/dl in female subjects; (3) systolic blood pressure ≥130 mmHg or diastolic blood pressure ≥ 85 mmHg; and (4) fasting plasma glucose ≥100 mg/dl^42^. Blood pressure was based on the average of two measurements.

### Genotyping

DNA was isolated from blood samples using a QIAamp DNA blood kit following the manufacturer’s instructions (Qiagen, Valencia, CA, USA). The quality of the isolated genomic DNA was evaluated using agarose gel electrophoresis, and the quantity was determined by spectrophotometry^43^. SNP genotyping was carried out using the custom Taiwan BioBank chips and run on the Axiom Genome-Wide Array Plate System (Affymetrix, Santa Clara, CA, USA). The SNP panel consisted of variants from the following genes: *APOA5*, *APOC1*, *BRAP*, *BUD13*, *CETP*, *LIPA*, *LPL*, *PLCG1*, and *ZPR1*.

### Statistical analysis

Categorical data were evaluated using the chi-square test. We conducted the Student’s t test to compare the difference in the means from two continuous variables. To estimate the association of the investigated SNP with MetS, we conducted a logistic regression analysis to evaluate the odds ratios (ORs) and their 95% confidence intervals (CIs), adjusting for covariates, including age, gender, smoking, alcohol consumption, and physical activity^44^. Furthermore, we estimated the association of the investigated SNP with individual components of MetS (as quantitative measures) by using linear regression analysis, adjusting for age, gender, smoking, alcohol consumption, and physical activity^45^. The genotype frequencies were assessed for Hardy-Weinberg equilibrium using a χ^2^ goodness-of-fit test with 1 degree of freedom (i.e. the number of genotypes minus the number of alleles). Multiple testing was adjusted by the Bonferroni correction. The criterion for significance was set at P < 0.05 for all tests. Data are presented as the mean ± standard deviation.

To investigate gene-gene and gene-environment interactions, we employed the generalized multifactor dimensionality reduction (GMDR) method^46^. We tested two-way up to five-way interactions using 10-fold cross-validation. The GMDR software provides some output parameters, including the testing accuracy and empirical P values, to assess each selected interaction. Moreover, we provided age, gender, smoking, alcohol consumption, and physical activity as covariates for gene-gene interaction models in our interaction analyses. We also prepared gender and age as covariates for gene-environment interaction models. Permutation testing obtains empirical P values of prediction accuracy as a benchmark based on 1,000 shuffles. In order to correct for multiple testing, we applied a conservative Bonferroni correction factor for the number of SNPs and environmental factors employed in the GMDR analysis.

Based on the effect sizes in this study, the power to detect significant associations was evaluated by QUANTO software ( http://biostats.usc.edu/Quanto.html).

## Additional Information

**How to cite this article**: Lin, E. *et al.* Association and interaction of *APOA5, BUD13, CETP, LIPA* and health-related behavior with metabolic syndrome in a Taiwanese population. *Sci. Rep.* **6**, 36830; doi: 10.1038/srep36830 (2016).

**Publisher’s note:** Springer Nature remains neutral with regard to jurisdictional claims in published maps and institutional affiliations.

## Supplementary Material

Supplementary Information

## Figures and Tables

**Table 1:** Demographic and clinical characteristics of study subjects.

Characteristic	Overall	MetS	No MetS	P value
No. of subjects, n	3000	533	2467	
Mean age ± SD, years	49.2 ± 11.0	53.3 ± 10.1	48.3 ± 10.9	<0.0001
Male, n/Female, n	1394/1606	257/276	1137/1330	0.371
High waist circumference^a^, n	1395	533	862	<0.0001
High triglyceride^b^, n	621	338	283	<0.0001
Low HDL^c^, n	713	339	374	<0.0001
High blood pressure^d^, n	694	290	404	<0.0001
High fasting glucose^e^, n	732	345	387	<0.0001
Current alcohol drinker, n	225	52	173	0.029
Current smoker, n	320	78	242	0.001
Physical activity, n	1759	309	1450	0.733

HDL = high-density lipoprotein cholesterol, MetS = metabolic syndrome, SD = standard deviation.

^a^Waist circumference ≥90 cm in male subjects, waist circumference ≥80 cm in female subjects.

^b^Triglyceride ≥150 mg/dl.

^c^HDL < 40 mg/dl in male subjects, HDL < 50 mg/dl in female subjects.

^d^Systolic blood pressure≥130 mmHg or diastolic blood pressure ≥85 mmHg.

^e^Fasting glucose ≥100 mg/dl.

**Table 2:** Odds ratio analysis with odds ratios after adjustment for covariates between the MetS and five SNPs including *APOA5* rs662799, *BUD13* rs11216129, *BUD13* rs623908, *CETP* rs820299, and *LIPA* rs1412444.

Gene	SNP	Case Allele and	Control Allele and	Additive			Recessive			Dominant		
Chr	Alleles	Genotype	Genotype	OR	95% CI	P	OR	95% CI	P	OR	95% CI	P
*APOA5*	rs662799	325/741	1303/3621	1.26	1.06-1.49	**0.0086**	1.47	1.06-2.04	0.0229	1.25	1.03-1.52	0.0218
11	C/T	54/217/262	173/957/1332									
*BUD13*	rs11216129	240/822	1319/3611	0.81	0.66-1.00	0.0532	0.74	0.49-1.12	0.1492	0.74	0.61-0.90	**0.0027**
11	A/C	29/182/320	177/965/1323									
*BUD13*	rs623908	295/767	1546/3372	0.90	0.75-1.06	0.2091	0.92	0.66-1.29	0.6357	0.75	0.61-0.90	**0.0027**
11	G/A	48/199/284	240/1066/1153									
*CETP*	rs820299	472/590	2020/2894	1.17	1.02-1.34	0.0211	1.47	1.16-1.86	**0.0015**	1.01	0.82-1.24	0.9387
16	G/A	118/236/177	416/1188/853									
*LIPA*	rs1412444	381/683	1577/3349	1.22	1.05-1.42	**0.0097**	1.41	1.06-1.88	0.0171	1.19	0.98-1.44	0.0826
10	T/C	74/233/225	260/1057/1146									

Chr = chromosome, CI = confidence interval, MetS = metabolic syndrome, OR = odds ratio.

Analysis was obtained after adjustment for covariates including age, gender, smoking, alcohol consumption, and physical activity. P values of <0.01 are shown in bold.

**Table 3:** Clinical characteristics of study subjects by genotypes in the *APOA5* rs662799, *BUD13* rs11216129, *BUD13* rs623908, *CETP* rs820299, and *LIPA* rs1412444 SNPs.

Characteristic	Genotype 1	Genotype 2	Genotype 3	P (Additive)	P (Recessive)	P (Dominant)
(1) *APOA5* rs662799	CC	CT	TT			
Waist circumference (cm)	56.5 ± 39.9	62.8 ± 37.3	61.7 ± 38.1	0.1116	0.0689	0.8496
Triglyceride (mg/dl)	157.0 ± 157.6	123.6 ± 89.6	106.3 ± 67.8	**6.25 × 10**^**−19**^	**6.72 × 10**^**−15**^	**1.73 × 10**^**−14**^
HDL (mg/dl)	51.07 ± 12.5	52.9 ± 13.1	54.8 ± 13.3	**2.50 × 10**^**−7**^	**2.45 × 10**^**−5**^	**2.11 × 10**^**−8**^
Systolic blood pressure (mmHg)	116.4 ± 17.6	114.9 ± 17.0	115.4 ± 16.7	0.2156	0.1674	0.9868
Diastolic blood pressure (mmHg)	70.9 ± 10.7	71.3 ± 11.0	71.7 ± 10.7	0.8443	0.9637	0.5186
Fasting glucose (mg/dl)	97.5 ± 22.1	97.1 ± 23.1	97.7 ± 20.9	0.8967	0.8418	0.8361
(2) *BUD13* rs11216129	AA	AC	CC			
Waist circumference (cm)	62.6 ± 37.3	60.0 ± 38.9	62.6 ± 37.4	0.9364	0.7327	0.0820
Triglyceride (mg/dl)	98.1 ± 59.8	108.2 ± 63.1	125.3 ± 102.9	**6.19 × 10**^**−6**^	0.0007	**1.80 × 10**^**−10**^
HDL (mg/dl)	56.6 ± 14.3	54.5 ± 12.8	53.0 ± 13.3	**2.88 × 10**^**−5**^	0.0006	**3.14 × 10**^**−6**^
Systolic blood pressure (mmHg)	114.9 ± 16.4	115.6 ± 17.0	115.1 ± 16.9	0.6987	0.6369	0.8509
Diastolic blood pressure (mmHg)	71.9 ± 10.5	71.7 ± 10.7	71.2 ± 10.9	0.6226	0.7011	0.5239
Fasting glucose (mg/dl)	95.6 ± 13.8	97.9 ± 22.6	97.4 ± 22.2	0.2086	0.1763	0.9021
(3) *BUD13* rs623908	GG	GA	AA			
Waist circumference (cm)	59.9 ± 38.8	60.9 ± 38.6	62.5 ± 37.3	0.2429	0.4101	0.1239
Triglyceride (mg/dl)	98.3 ± 59.4	110.6 ± 67.9	126.0 ± 105.0	**1.66 × 10**^**−7**^	**9.75 × 10**^**−5**^	**4.32 × 10**^**−10**^
HDL (mg/dl)	56.04 ± 14.4	54.06 ± 12.7	53.06 ± 13.4	**5.14 × 10**^**−5**^	0.0008	**9.40 × 10**^**−5**^
Systolic blood pressure (mmHg)	114.7 ± 16.3	115.7 ± 16.9	114.9 ± 17.0	0.8652	0.7711	0.7794
Diastolic blood pressure (mmHg)	71.7 ± 10.5	71.8 ± 10.7	71.1 ± 11.0	0.5012	0.6209	0.4082
Fasting glucose (mg/dl)	96.0 ± 16.0	97.8 ± 22.2	97.5 ± 22.7	0.2846	0.2939	0.5876
(4) *CETP* rs820299	GG	GA	AA			
Waist circumference (cm)	60.2 ± 38.7	62.5 ± 38.0	61.3 ± 37.5	0.7405	0.3801	0.5074
Triglyceride (mg/dl)	119.3 ± 78.5	117.0 ± 92.6	115.3 ± 84.8	0.2492	0.2728	0.4642
HDL (mg/dl)	53.08 ± 13.6	53.67 ± 13.1	54.34 ± 13.1	0.0081	0.0339	0.0214
Systolic blood pressure (mmHg)	115.6 ± 16.7	115.9 ± 17.3	114.3 ± 16.4	0.0487	0.1538	0.0512
Diastolic blood pressure (mmHg)	71.6 ± 11.2	71.6 ± 10.9	71.4 ± 10.5	0.2542	0.3229	0.3820
Fasting glucose (mg/dl)	97.4 ± 21.1	98.0 ± 22.5	96.8 ± 21.5	0.5010	0.7046	0.4047
(5) *LIPA* rs1412444	TT	TC	CC			
Waist circumference (cm)	64.1 ± 37.1	61.0 ± 38.2	61.6 ± 38.1	0.3757	0.2571	0.8255
Triglyceride (mg/dl)	122.6 ± 74.0	117.7 ± 95.8	114.6 ± 82.4	0.1922	0.2323	0.3659
HDL (mg/dl)	51.9 ± 12.6	53.64 ± 13.3	54.39 ± 13.3	0.0032	0.0042	0.1083
Systolic blood pressure (mmHg)	115.0 ± 16.3	115.9 ± 16.8	114.8 ± 17.1	0.8049	0.8659	0.2318
Diastolic blood pressure (mmHg)	71.5 ± 10.7	71.9 ± 10.6	71.1 ± 11.0	0.7929	0.8811	0.2327
Fasting glucose (mg/dl)	100.4 ± 27.5	97.8 ± 22.4	96.4 ± 19.6	0.0021	0.0048	0.0343

HDL = high-density lipoprotein cholesterol. Analysis was obtained after adjustment for covariates including age, gender, smoking, alcohol consumption, and physical activity. P values of < 0.0006 (Bonferroni correction: 0.05/82) are shown in bold.

**Table 4:** Gene-gene interaction models identified by the GMDR method with adjustment for age, gender, smoking, alcohol consumption, and physical activity.

Phenotype	Best interaction model	Testing accuracy (%)	P value
(a) Two-way interaction models			
MetS	*CETP* rs820299, *LIPA* rs1412444	54.19	**0.005**
High waist circumference^a^	*BUD13* rs623908, *CETP* rs820299	51.96	0.056
High triglyceride^b^	*APOA5* rs662799, *LIPA* rs1412444	56.69	**<0.001**
Low HDL^c^	*APOA5* rs662799, *CETP* rs820299	55.90	**< 0.001**
High blood pressure^d^	*APOA5* rs662799, *CETP* rs820299	51.45	0.205
High fasting glucose^e^	*BUD13* rs11216129, *LIPA* rs1412444	53.31	**0.007**
(b) Three-way interaction models			
MetS	*BUD13* rs623908, *CETP* rs820299, *LIPA* rs1412444	55.59	**0.001**
High waist circumference^a^	*BUD13* rs623908, *CETP* rs820299, *LIPA* rs1412444	49.55	0.618
High triglyceride^b^	*APOA5* rs662799, *BUD13* rs623908, *LIPA* rs1412444	59.10	**< 0.001**
Low HDL^c^	*APOA5* rs662799, *CETP* rs820299, *LIPA* rs1412444	54.84	**< 0.001**
High blood pressure^d^	*APOA5* rs662799, *CETP* rs820299, *LIPA* rs1412444	51.74	0.167
High fasting glucose^e^	*BUD13* rs11216129, *CETP* rs820299, *LIPA* rs1412444	54.34	**0.004**
(c) Four-way interaction models			
MetS	*APOA5* rs662799, *BUD13* rs623908, *CETP* rs820299, *LIPA* rs1412444	53.99	0.012
High waist circumference^a^	*APOA5* rs662799, *BUD13* rs623908, *CETP* rs820299, *LIPA* rs1412444	50.49	0.374
High triglyceride^b^	*APOA5* rs662799, *BUD13* rs623908, *CETP* rs820299, *LIPA* rs1412444	58.30	**< 0.001**
Low HDL^c^	*APOA5* rs662799, *BUD13* rs623908, *CETP* rs820299, *LIPA* rs1412444	56.52	**< 0.001**
High blood pressure^d^	*APOA5* rs662799, *BUD13* rs623908, *CETP* rs820299, *LIPA* rs1412444	51.93	0.135
High fasting glucose^e^	*APOA5* rs662799, *BUD13* rs11216129, *CETP* rs820299, *LIPA* rs1412444	51.50	0.195
(d) Five-way interaction models			
MetS	*APOA5* rs662799, *BUD13* rs11216129, *BUD13* rs623908, *CETP* rs820299, *LIPA* rs1412444	52.47	0.093
High waist circumference^a^	*APOA5* rs662799, *BUD13* rs11216129, *BUD13* rs623908, *CETP* rs820299, *LIPA* rs1412444	50.64	0.334
High triglyceride^b^	*APOA5* rs662799, *BUD13* rs11216129, *BUD13* rs623908, *CETP* rs820299, *LIPA* rs1412444	58.03	**< 0.001**
Low HDL^c^	*APOA5* rs662799, *BUD13* rs11216129, *BUD13* rs623908, *CETP* rs820299, *LIPA* rs1412444	55.53	**0.001**
High blood pressure^d^	*APOA5* rs662799, *BUD13* rs11216129, *BUD13* rs623908, *CETP* rs820299, *LIPA* rs1412444	50.94	0.294
High fasting glucose^e^	*APOA5* rs662799, *BUD13* rs11216129, *BUD13* rs623908, *CETP* rs820299, *LIPA* rs1412444	51.75	0.151

GMDR = generalized multifactor dimensionality reduction, HDL = high-density lipoprotein cholesterol, MetS = metabolic syndrome.

P value was based on 1,000 permutations. Analysis was obtained after adjustment for covariates including age, gender, smoking, alcohol consumption, and physical activity. P values of < 0.01 (Bonferroni correction: 0.05/5) are shown in bold.

^a^Waist circumference ≥90 cm in male subjects, waist circumference ≥80 cm in female subjects.

^b^Triglyceride ≥150 mg/dl.

^c^HDL < 40 mg/dl in male subjects, HDL < 50 mg/dl in female subjects.

^d^Systolic blood pressure ≥130 mmHg or diastolic blood pressure ≥85 mmHg.

^e^Fasting glucose ≥100 mg/dl.

**Table 5:** Gene-environment interaction models identified by the GMDR method with adjustment for age and gender.

Phenotype	Best interaction model	Testing accuracy (%)	P value
(a) Two-way interaction models			
MetS	*BUD13* rs623908, smoking	55.36	**< 0.001**
High waist circumference^a^	*CETP* rs820299, physical activity	53.93	**< 0.001**
High triglyceride^b^	*APOA5* rs662799, smoking	58.55	**< 0.001**
Low HDL^c^	*APOA5* rs662799, smoking	55.08	**< 0.001**
High blood pressure^d^	*CETP* rs820299, physical activity	53.41	0.012
High fasting glucose^e^	*LIPA* rs1412444, alcohol consumption	51.54	0.145
(b) Three-way interaction models			
MetS	*BUD13* rs623908, *CETP* rs820299, smoking	55.24	**< 0.001**
High waist circumference^a^	*BUD13* rs623908, *CETP* rs820299, physical activity	53.85	**0.002**
High triglyceride^b^	*APOA5* rs662799, *LIPA* rs1412444, smoking	57.90	**< 0.001**
Low HDL^c^	*APOA5* rs662799, *LIPA* rs1412444, smoking	56.56	**< 0.001**
High blood pressure^d^	*CETP* rs820299, *LIPA* rs1412444, physical activity	51.40	0.221
High fasting glucose^e^	*BUD13* rs11216129, *LIPA* rs1412444, alcohol consumption	53.91	**0.004**
(c) Four-way interaction models			
MetS	*BUD13* rs623908, *CETP* rs820299, *LIPA* rs1412444, smoking	56.02	**<0.001**
High waist circumference^a^	*BUD13* rs623908, *CETP* rs820299, *LIPA* rs1412444, physical activity	52.37	0.045
High triglyceride^b^	*APOA5* rs662799, *CETP* rs820299, *LIPA* rs1412444, smoking	59.76	**< 0.001**
Low HDL^c^	*APOA5* rs662799, *CETP* rs820299, *LIPA* rs1412444, smoking	56.60	**< 0.001**
High blood pressure^d^	*BUD13* rs623908, *CETP* rs820299, *LIPA* rs1412444, physical activity	52.27	0.094
High fasting glucose^e^	*BUD13* rs11216129, *CETP* rs820299, *LIPA* rs1412444, alcohol consumption	53.67	0.007
(d) Five-way interaction models			
MetS	*BUD13* rs623908, *CETP* rs820299, *LIPA* rs1412444, smoking, physical activity	55.66	**< 0.001**
High waist circumference^a^	*BUD13* rs623908, *CETP* rs820299, *LIPA* rs1412444, smoking, physical activity	53.13	0.012
High triglyceride^b^	*APOA5* rs662799, *BUD13* rs623908, *CETP* rs820299, *LIPA* rs1412444, smoking	58.58	**< 0.001**
Low HDL^c^	*APOA5* rs662799, *BUD13* rs623908, *CETP* rs820299, *LIPA* rs1412444, smoking	57.76	**< 0.001**
High blood pressure^d^	*APOA5* rs662799, *BUD13* rs623908, *CETP* rs820299, *LIPA* rs1412444, physical activity	50.46	0.389
High fasting glucose^e^	*APOA5* rs662799, *BUD13* rs11216129, *CETP* rs820299, *LIPA* rs1412444, physical activity	51.87	0.121

GMDR = generalized multifactor dimensionality reduction, HDL = high-density lipoprotein cholesterol, MetS = metabolic syndrome.

P value was based on 1,000 permutations. Analysis was obtained after adjustment for covariates including age and gender.

P values of <0.006 (Bonferroni correction: 0.05/8) are shown in bold.

^a^Waist circumference ≥90 cm in male subjects, waist circumference ≥80 cm in female subjects.

^b^Triglyceride ≥150 mg/dl.

^c^HDL< 40 mg/dl in male subjects, HDL < 50 mg/dl in female subjects.

^d^Systolic blood pressure ≥130 mmHg or diastolic blood pressure ≥85 mmHg.

^e^Fasting glucose ≥100 mg/dl.
