# Supplementary material for: Association and interaction of APOA5, BUD13, CETP, LIPA and health-related behavior with metabolic syndrome in a Taiwanese population
Source: Sci Rep. 2016 Nov 9;6:36830. doi: 10.1038/srep36830 (PMC5101796; doi:10.1038/srep36830)
Supplement: Supplementary Information [file srep36830-s1.pdf]

Supplementary Information for

Association and interaction of *APOA5*, *BUD13*, *CETP*, *LIPA* and health-related behavior with metabolic syndrome in a Taiwanese population

Eugene Lin[1,2,3], Po-Hsiu Kuo[4], Yu-Li Liu[5], Albert C. Yang[6,7], Chung-Feng Kao[8], Shih-Jen Tsai[6,7]

[1]Graduate Institute of Biomedical Sciences, China Medical University, Taichung, Taiwan

[2]Vita Genomics, Inc., Taipei, Taiwan

[3]TickleFish Systems Corporation, Seattle, WA, USA

[4]Department of Public Health, Institute of Epidemiology and Preventive Medicine, National Taiwan University, Taipei, Taiwan

[5]Center for Neuropsychiatric Research, National Health Research Institutes, Miaoli County, Taiwan

[6]Department of Psychiatry, Taipei Veterans General Hospital, Taipei, Taiwan

[7]Division of Psychiatry, National Yang-Ming University, Taipei, Taiwan

[8]Department of Agronomy, College of Agriculture & Natural Resources, National Chung

Hsing University, Taichung, Taiwan

Corresponding author:

Dr. Eugene Lin, Vita Genomics, Inc, 7 Fl., No. 6, Sec. 1, Jung-Shing Road,

Wugu Shiang, Taipei, Taiwan

E-mail: eugene.lin@vitagenomics.com

Phone: (+ 886) 2-8976-9123 ext 7751

Fax: (+ 886) 2-8976-9523

or

Dr. Shih-Jen Tsai, Department of Psychiatry, Taipei Veterans General Hospital, No. 201, Shih-Pai Road, Sec. 2, 11217, Taipei, Taiwan

E-mail: tsai610913@gmail.com

Phone: 886-2-2875 7027, ext. 276

Fax: 886-2-2872 5643

Supplementary Table S1. Odds ratio analysis after adjustment for covariates between the MetS and 82 SNPs in the *APOA5, APOC1, BRAP, BUD13, CETP, LIPA, LPL, PLCG1,* and *ZPR1* genes.

Gene	CHR	SNP	Alleles	P (Dominant)	P (Additive)	P (Recessive)
APOA5	11	rs2266788	G/A	0.6794	0.7721	0.8111
		rs662799	C/T	**0.0218**	**0.0086**	**0.0229**
APOC1	19	rs445925	A/G	0.9888	0.0604	0.0581
		rs4420638	G/A	0.2980	0.6755	0.7119
BRAP	12	rs3742001	C/T	0.5105	0.5402	0.5228
		rs3782886	C/T	0.9824	0.5829	0.5342
		rs12427276	A/G	0.1719	0.7309	0.9831
BUD13	11	rs11556024	A/G	0.4681	0.6404	0.6623
		rs77684111	A/G	0.4973	0.3052	0.3164
		rs11216129	A/C	**0.0027**	0.0532	0.1492
		rs2849179	T/C	**0.0318**	0.3695	0.9217
		rs180327	C/T	0.5484	0.5719	0.6540
		rs180326	G/T	0.5924	0.8338	0.7603
		rs2075295	C/T	0.1354	0.0690	0.1299
		rs3741301	C/T	0.3437	0.5343	0.6648
		rs3741300	A/G	**0.0218**	0.3305	0.9138
		rs10488698	A/G	0.4433	0.7014	0.6847
		rs10790162	A/G	0.6475	0.9216	0.9745
		rs1263149	G/A	0.0900	0.1032	0.2772
		rs623908	G/A	**0.0027**	0.2091	0.6357
CETP	16	rs4783961	A/G	0.2905	0.2227	0.2722
		rs1800775	C/A	0.9061	0.1253	**0.0148**
		rs711752	A/G	**0.0168**	0.2400	0.8672

		rs1864163	A/G	0.3282	0.9689	0.9131
		rs7203984	C/A	0.8377	0.6332	0.6351
		rs11508026	T/C	**0.0173**	0.2607	0.5626
		rs820299	G/A	0.9387	**0.0211**	**0.0015**
		rs9939224	T/G	0.3042	0.8272	0.7595
		rs1532624	A/C	**0.0138**	0.5243	0.9856
		rs7499892	T/C	**0.0134**	0.3948	0.5287
		rs289715	A/T	0.8751	0.9693	0.9852
		rs75511668	C/T	0.3164	0.0921	0.1032
		rs289719	T/C	0.0905	0.5607	0.8849
		rs4784744	G/A	**0.0205**	**0.0492**	0.2325
		rs1800774	T/C	0.0730	**0.0474**	0.0572
		rs5882	G/A	0.5158	0.9762	0.5931
LIPA	10	rs10749600	G/A	0.1530	0.0593	0.0675
		rs10509568	A/G	0.1248	0.9055	0.8956
		rs6586175	A/G	0.2912	0.2008	0.2187
		rs1556478	C/T	0.3575	0.1622	0.1822
		rs2297472	G/A	0.4766	0.2484	0.2715
		rs2254747	C/T	0.6932	0.0881	0.0851
		rs2254670	C/T	0.1714	0.0506	0.0566
		rs2071510	G/T	0.4131	0.9431	0.6198
		rs11203042	C/T	0.5724	0.6534	0.8244
		rs1041389	T/C	0.8461	0.5470	0.5148
		rs885561	C/T	0.9368	0.8186	0.7183
		rs1029074	G/A	0.3334	0.0808	0.0712
		rs1412444	T/C	0.0826	**0.0097**	**0.0171**
		rs2246828	A/G	0.3550	0.0926	0.1009
		rs928415	A/G	0.8885	0.9982	0.9982
		rs2250781	A/C	0.0605	0.0746	0.2512
LPL	8	rs3779788	T/C	0.0786	0.2992	0.3746
		rs74377536	A/C	0.9068	0.5091	0.4530
		rs73667472	C/T	0.2233	0.7957	0.7671

		rs249	C/T	0.3525	0.6624	0.6781
		rs253	T/C	0.2446	0.3742	0.5769
		rs254	G/C	0.1749	0.0800	0.1034
		rs268	G/A	0.8887	0.8481	0.8420
		rs270	A/C	0.5146	0.8274	0.8651
		rs271	A/G	0.1886	**0.0318**	**0.0395**
		rs281	T/A	0.6318	0.4198	0.3167
		rs283	T/C	0.7962	0.8774	0.8670
		rs295	C/A	0.1193	**0.0351**	**0.0463**
		rs301	C/T	0.1274	0.0666	0.0875
		rs316	A/C	0.9641	0.8299	0.8263
		rs319	C/A	0.7126	0.2166	0.1871
		rs325	C/T	**0.0131**	0.1124	0.1350
		rs326	G/A	0.1216	**0.0483**	0.0636
		rs327	G/T	0.0900	0.0658	0.0913
		rs328	G/C	**0.0119**	0.1113	0.1342
		rs10099160	G/T	0.9274	0.2694	0.2563
		rs1059507	T/C	0.8759	0.9110	0.9157
		rs13702	C/T	0.1200	0.0911	0.1199
		rs15285	T/C	0.1300	0.0644	0.0835
PLCG1	20	rs77440706	C/T	0.7239	0.2559	0.2579
		rs2066906	A/G	0.6052	0.1720	0.1045
ZPR1	11	rs964184	G/C	0.6671	0.8247	0.8695
		rs6589566	G/A	0.6316	0.5139	0.5400
		rs2075290	C/T	0.8816	0.7943	0.7612
		rs603446	T/C	0.4093	0.6893	0.8240
		rs3741298	C/T	0.6640	0.2783	0.2532

Chr = chromosome, MetS = metabolic syndrome.

Analysis was obtained after adjustment for covariates including age, gender, smoking, alcohol consumption, and physical activity. P values of < 0.05 are shown in bold.

Supplementary Table S2. Pairwise LD measures for SNPs with strong LD ($r^2 > 0.8$).

Gene	CHR	SNP 1	SNP 2	r^2
BUD13	11	rs11556024	rs77684111	0.99
		rs2849179	rs3741300	0.97
		rs180326	rs10790162	0.91
		rs2075295	rs1263149	0.94
CETP	16	rs1864163	rs9939224	0.88
		rs11508026	rs1532624	0.91
LIPA	10	rs10749600	rs6586175	0.84
		rs10749600	rs2254670	0.87
		rs6586175	rs2254670	0.86
		rs1556478	rs2297472	0.82
		rs11203042	rs885561	0.88
LPL	8	rs73667472	rs249	0.89
		rs254	rs271	0.99
		rs283	rs319	0.80
		rs295	rs301	0.99
		rs295	rs326	0.99
		rs295	rs327	0.98
		rs301	rs326	0.99
		rs301	rs327	0.98
		rs301	rs13702	0.95
		rs316	rs1059507	0.93
		rs319	rs10099160	0.92
		rs325	rs328	1.00
		rs326	rs327	0.99
		rs326	rs13702	0.96
		rs326	rs15285	0.96
		rs327	rs13702	0.95

		rs327	rs15285	0.95
		rs13702	rs15285	1.00
ZPR1	11	rs964184	rs6589566	0.97
		rs964184	rs2075290	0.91
		rs6589566	rs2075290	0.88

Chr = chromosome, LD = linkage disequilibrium, MetS = metabolic syndrome.

Supplementary Table S3. Multivariable logistic regression analysis for the two-way gene-environment interaction models.

Two-way interaction model	OR	95% CI	P value[b]
(a) MetS			
Non-smokers with *BUD13* rs623908 (AA genotpye)[a]	1		
Smokers with *BUD13* rs623908 (AA genotpye)	1.53	1.00-2.33	0.0516
Non-smokers with *BUD13* rs623908 (GA+GG genotpye)	0.71	0.58-0.87	0.0012
Smokers with *BUD13* rs623908 (GA+GG genotpye)	1.61	1.09-2.39	0.0175
(b) High waist circumference[c]			
CETP rs820299 (AA+GA genotpye) and high physical activity[a]	1		
CETP rs820299 (AA+GA genotpye) and low physical activity	1.20	1.01-1.43	0.0410
CETP rs820299 (GG genotpye) and high physical activity	1.25	0.92-1.70	0.1458
CETP rs820299 (GG genotpye) and low physical activity	1.44	1.10-1.87	0.0073
(c) High triglyceride[d]			
Non-smokers with *APOA5 rs662799* (TT genotpye)[a]	1		
Smokers with *APOA5 rs662799* (TT genotpye)	1.81	1.26-2.62	0.0014
Non-smokers with *APOA5 rs662799* (CT+CC genotpye)	1.93	1.58-2.36	1.0×10^{-10}
Smokers with	3.42	2.35-4.97	1.3×10^{-10}

APOA5 rs662799 (CT+CC genotpye)			
(d) Low HDL[e]			
Non-smokers with *APOA5 rs662799* (TT genotpye)[a]	1		
Smokers with *APOA5 rs662799* (TT genotpye)	1.99	1.37-2.90	0.0003
Non-smokers with *APOA5 rs662799* (CT+CC genotpye)	1.49	1.25-1.79	$1.5x10^{-5}$
Smokers with *APOA5 rs662799* (CT+CC genotpye)	2.62	1.77-3.86	$1.3x10^{-6}$

CI = confidence interval, MetS = metabolic syndrome, OR = odds ratio.

[a] Reference.

[b] Versus reference. Analysis was obtained after adjustment for covariates including age and gender.

[c] Waist circumference ≥ 90 cm in male subjects, waist circumference ≥ 80 cm in female subjects.

[d] Triglyceride ≥ 150 mg/dl.

[e] HDL< 40 mg/dl in male subjects, HDL < 50 mg/dl in female subjects.
